# Willingness, Attitude, and Associated Factors towards Adverse Drug Reaction Reporting among Healthcare Providers in Mizan Tepi University Teaching Hospital, Southwest Ethiopia

**DOI:** 10.1155/2022/1368624

**Published:** 2022-12-06

**Authors:** Jafer Siraj, Miftah Shafi, Fikadu Ejeta, Diriba Feyisa, Oliyad Kebede, Suleman Hassen

**Affiliations:** ^1^Department of Pharmacy, School of Applied Natural Science, Adama Science and Technology University, Adama, Ethiopia; ^2^School of Pharmacy, College of Medicine and Health Sciences, Mizan-Tepi University, Mizan-Aman, Ethiopia; ^3^School of Medicine, College of Health Sciences, Selale University, Fiche, Ethiopia; ^4^School of Medicine, College of Medicine and Health Sciences, Mizan-Tepi University, Mizan-Aman, Ethiopia

## Abstract

**Background:**

An adverse drug reaction (ADR) is harm that arises from the use of a drug. Adverse drug reactions have a huge impact on the health system since they result in drug-related morbidity and mortality as well as indirect costs such as loss of productivity at work. Reporting of adverse drug reactions to a relevant authority is one of the methods of enhancing medication safety; however, underreporting of adverse drug reactions by health workers is a major challenge in enhancing medicines safety. The aim of this study was to assess the willingness, attitude, and associated factors of healthcare providers towards ADRs reporting at Mizan Tepi University Teaching Hospital (MTUTH).

**Methods:**

A cross-sectional mixed method was conducted among healthcare providers working in Mizan Tepi University Teaching Hospital from August to September 2021. A total of 190 healthcare providers and five key informants were participated in the study. The quantitative data were collected using self-administered structured questionnaires, and for the qualitative study, data from the key informant interviews were collected using a semistructured questionnaire containing open-ended questions. The collected data were coded, entered, and analyzed using a Statistical Package for Social Sciences (SPSS, version 21). Furthermore, the logistic regression model was fitted to see the association between attitude items and demographic characteristics. Variables with *P* value <0.05 were considered as statistically significant. Analysis of the qualitative data was done by sorting the data into categories and examining the emerging themes.

**Results:**

The majority of individuals in the study (81.6%) were willing to report adverse drug reactions. High percent (57.9%) of the study participants knew how to report ADR to responsible body and 66 (34.7%) of the study participants believe that ADR reporting is the responsibility of all healthcare professionals. 86 (45.3%) of respondents encountered ADR cases during their professional career. It was found that the majority of participants (53.7%) had a positive attitude toward ADR reporting. The professional distribution and work experiences of healthcare professionals had a significant impact on their attitude toward ADR reporting (*P* < 0.05).

**Conclusion:**

A large percentage of study subjects were willing to report adverse drug reactions to the appropriate authorities. The majority of healthcare providers were found to have a good attitude towards ADR reporting. Professional distribution and work experiences were significantly associated with attitude of healthcare providers towards ADR reporting. In order to improve the ADR reporting practices of the healthcare professionals and increase the ADR reporting load at the national level, the national regulatory body should collaborate with health facilities.

## 1. Introduction

Adverse drug reactions (ADRs) are an imperative root of death and illness around the universe. ADRs are hazardous and accidental pharmaceutical reactions that occur at optimum levels utilized in persons for disease prevention, diagnosis, therapy, or physiological activity adjustment [1]. ADRs are defined by Edwards and Aronson as “a significantly harmful or unpleasant reaction resulting from an intervention related to the use of a therapeutic item, which predicts vulnerability from future administration and allows prevention or specific treatment, or dosage regime modification, or withdrawal of the medication” [2]. A side effect that develops while a patient is taking medication but is not always related to it is called an adverse drug event. Therefore, adverse drug events include harm caused by the utilization of medication (adverse drug reaction) as well as harm from the use of a product [2, 3]. It may or may not have a causal relation with the drug administered. Adverse events are both preventable and unpreventable. Medication errors account for about 25% of adverse drug events [4].

One component of pharmacovigilance programs that has not gotten much attention is how adverse drug reactions are reported. After they are released onto the market, drugs are likely to experience unexpected adverse effects [5]. Healthcare personnel with direct patient contact, like pharmacists, doctors, and nurses, are best placed to spot such effects [6]. Healthcare professionals or the general public can report ADRs. Healthcare professionals are mandated to document adverse medication responses in several nations [7, 8].

The most important step in improving drug safety is reporting adverse events to appropriate national regulatory bodies. According to the severity of adverse drug responses, the regulatory authority then assesses the reports given by the various players, using the results to decide whether or not the drug items should be taken off the market. The major drawback of this approach is that ADRs go unreported and without proper attention [9]. Most nations have healthcare professionals who record adverse drug reactions rather than patients and clients, which might lead to underreporting and neglect of adverse drug reactions [10]. Studies show that a number of elements related to healthcare providers, the healthcare system, and patients have an impact on how ADRs are reported to the drug regulatory agency. These studies found a high relationship between ADR reporting practices and the age, professional role, work experience, knowledge, and attitudes of healthcare professionals [11–13].

ADR reporting remains poor in Ethiopia despite the Ethiopian Food and Drug Administration (EFDA) establishing a pharmacovigilance program. The identification and monitoring of adverse drug reactions have been given top priority by the Ethiopian federal government [7]. Analysis and reporting of potential adverse medication reactions to Ethiopia's national regulatory agency is the responsibility of healthcare practitioners. The national drug regulatory agency must conduct extra quality assurance and safety control activities as a result of this investigation [7, 14]. Additionally, it is strongly urged that everyone and the general public understand the significance of informing the proper authorities, such as medical professionals and the Ethiopian Food and Drug Administration, of any suspected or actual adverse drug reactions [1, 15]. The majority of ADR in Ethiopia is, however, reported by healthcare professionals rather than the public.

Under the former Ethiopian Food, Medicine, and Healthcare Administration and Control Authority (EFMHACA), now known as the Ethiopian Food and Drug Authority (EFDA), Ethiopia launched its pharmacovigilance program. Ethiopia is a recognized participant in the WHO Uppsala International Drug Monitoring Center (UMC) [16]. The authority was required to conduct postmarketing surveillance (PMS) activities by EFDA proclamation no. 1112/2019, Article 4 subarticle 9, in order to ensure the safety, efficacy, and quality of medicines as well as appropriate legal measures for patient safety and better treatment outcomes for products marketed in the nation [17]. Healthcare practitioners have been urged to report ADRs using the ADR reporting form, which has been made available throughout all medical facilities, since the EFDA spontaneous Adverse Drug Reaction reporting program was launched in 2002. The authority has created pharmacovigilance guidelines and is required to look into safety concerns and take appropriate action to avoid and limit medication-related damage [14].

Studies carried out across Ethiopia indicate that healthcare professionals do not consistently disclose ADRs. Additionally, those studies made the recommendation that relevant entities aid in educating healthcare professionals and the general public about ADR detection and reporting systems [13, 18]. The ADR cases listed on patients' cards and the ADR reports filed to the Ethiopian Food and Drug Administration differ. The practice of reporting adverse medication reactions is unknown at Mizan Tepi University Teaching Hospital (MTUTH), the causes are not thoroughly investigated, and there is a shortage of information among healthcare professionals. This shows that healthcare professionals in the research area may not be paying enough attention to ADR reporting and that there may be a knowledge gap surrounding it. As a result, the current study will provide adequate details regarding the ADR reporting procedures used by healthcare professionals and will aid study participants in developing better ADR reporting practices. Additionally, give crucial information to the national regulatory authorities. Therefore, the aim of this study was to examine the willingness, attitude, and associated factors of healthcare professionals to report ADRs at Mizan Tepi University Teaching Hospital (MTUTH).

## 2. Methods

### 2.1. Study Design and Study Setting

A cross-sectional mixed method was conducted among healthcare providers working in Mizan Tepi University Teaching Hospital from August to September 2021. This hospital is located in Mizan-Aman town, Southwest Ethiopia. It offers both inpatient and outpatient services and has many wards, including those for pediatrics, internal medicine, surgery, gynecology, and ambulatory care. The hospital also provides patients with access to four pharmacies: an outpatient pharmacy, an antiretroviral therapy pharmacy, an emergency pharmacy, and an inpatient pharmacy. With a total of 110 beds, it served 2 million people from four zones, including Bench Sheko, Kefa, Sheka, and Majang, as referral centers. On average, 350 patients come to the hospital each day for various services. More than 363 people worked at the hospital, including doctors, pharmacists, lab technicians, nurses, administrative employees, and support workers.

### 2.2. Sample Size and Eligibility Criteria

A total of 190 healthcare professionals from Mizan Tepi University Teaching Hospital participated in the study. Participants in the study were healthcare professionals who were actively involved in clinical patient care and in a position to identify adverse drug reactions, such as nurses, doctors, public health officers, pharmacists, anesthesiologists, and psychologists. However, healthcare professionals who refused to take part in the study were not included.

The sample size was chosen beforehand in order to select important informants from related divisions such psychiatry, pharmacy, doctors, nurses, and public health officer for the qualitative study. Through the use of a focused sample method, the key informants were selected. Five key informants were identified from each department to participate in a thorough interview. The key informants were selected based on their extensive knowledge on adverse drug reaction reporting.

### 2.3. Data Collection Tools and Procedures

Self-administered structured questionnaires that were constructed by evaluating previous surveys with various revisions were used to collect the quantitative data [12, 13, 18]. The questionnaire asked about the basic demographics of the study participants as well as the willingness, knowledge, attitude, and practices of healthcare professionals with regard to reporting ADRs. Before the actual data collection, the questionnaire was pretested with 19 healthcare professionals to ensure the validity of the study of the instruments and respondent's grasp of the questions. On the basis of the feedback received from the pilot testing, minor adjustments were made. Data from the pilot research were not included in the analysis. Cronbach's alpha coefficient, which measures internal consistency, was calculated and found to be 0.79, which is within the acceptable range. To aid in the data collection procedure, two qualified pharmacy experts were hired. The supervisors examined the collected data for completeness and consistency.

For the qualitative investigation, the information from the key informant was gathered using a semistructured questionnaire with open-ended questions. The inquiries necessary to gain knowledge of the elements inside the healthcare facility encouraged and discouraged the reporting of adverse medication reactions by healthcare professionals. To further investigate the potential institutional elements that may affect healthcare professionals' reporting of ADRs, the authors of this research performed in-depth face-to-face interviews with the chosen key informants. The interviewee's responses to the questions were noticed and recorded.

### 2.4. Data Analysis

Statistical Package for Social Sciences (SPSS, version 21) was used to code, enter, and analyze the data that had been gathered. Demographic characteristics, willingness, knowledge, attitude, and practice of the study participants with regard to ADRs reporting were summarized using descriptive statistics such as frequency, percentage, and mean. Ten items graded as agree, neutral, disagree, or strongly disagree on a five-point Likert scale were used to gauge the attitude of the healthcare providers. For all questions, an answer of “Strongly agree” received a score of 5, “Agree” received a score of 4, “Neutral” received a score of 3, “Disagree” received a score of 2, and “Strongly disagree” received a score of 1; negatively worded questions received a lower score. The mean score was used to determine the overall attitude level. Participants were categorized as having a positive attitude if their scores were greater than or equal to the mean, while participants with lower scores were categorized as having a negative attitude. The logistic regression model was additionally built to examine the relationship between attitude items and demographic traits. Variables having a *P* value 0.25 from the results of the binary logistic regression were added to the multivariate logistic regression, and variables with a *P* value 0.05 were deemed statistically significant.

Nonverbatim transcription was used to translate the interview notes and audio recordings for the qualitative investigation. Before data synthesis and report writing, four seasoned reviewers examined the transcript and offered feedback for the analysis. The transcribed scripts were carefully reviewed to find the major themes, and the information was then thematically combined. Thematic analysis was employed during the manual study of the data. One of the recordings was translated and transcribed by a bilingual specialist, and the results were compared with the original material to ensure that the translation was accurate. To ensure the accuracy of the transcripts and interpretation, the key informants were also informed of the study's findings.

## 3. Results

### 3.1. Sociodemographic Characteristics of Healthcare Providers

A total of 190 healthcare providers were participated in the study. Among the healthcare providers participated in the study, about 109 (57.4%) were male. Less than half (41.6%) of the respondents were in between 28 and 32 years age group. The majority of healthcare professionals (57.9%) have 1–3 years of job experience. Additionally, nurses made up 56.8% of the participating healthcare professionals, followed by doctors and pharmacists, who made up 22.6% and 16.8% of the total, respectively (Table 1).

### 3.2. Willingness of Healthcare Providers towards ADR Reporting

As shown in Table 2, a significant majority of survey participants (81.6%) were willing to report adverse drug reactions to the relevant organizations, while the remaining 18.4% of healthcare professionals were not. The two main factors that contributed to a lack of willingness to report ADRs in the study area were a lack of an ADR reporting format in the healthcare facility and a lack of knowledge about the ADRs reporting system which accounted of 48.6% and 25.7%, respectively.

### 3.3. Knowledge of Healthcare Providers towards ADR Reporting

Of the 190 survey respondents, 73% were unaware of the Ethiopian ADR reporting mechanism. Over half (48.9%) of healthcare professionals were not aware that there were national ADR monitoring criteria in place. The majority of study participants (57.9%) are aware of the proper channels to use when reporting ADR. ADR complaints should be monitored by the Ministry of Health, according to about 38.4% of respondents, followed with the EFDA and Ethiopian Pharmaceutical Association garnered 29.9% and 10% of the vote, respectively. 62.63 percent of those who took the survey had never heard of the ADR reporting form. About 66 study participants (34.7%) believe that reporting of ADRs is the responsibility of all healthcare providers (Table 3).

### 3.4. Healthcare Providers Practices towards ADR Reporting

86 (45.3%) of the 190 research participants who took part in the survey reported having dealt with ADR cases in the course of their professional careers. 53 (61.6%) of healthcare professionals recorded ADR cases in patients' medical records, while only 57 (66.28%) reported ADR cases to the proper authorities throughout their professional careers. More than half of the respondents (54.39%) indicated that they had been reported 2-3 times for ADR incidents throughout their professional careers. 60.5% of the medical professionals in the morning session had ever encountered ADR matters (Table 4).

### 3.5. Attitude of Healthcare Providers towards ADR Reporting

As seen in Table 5, out of the enrolled research respondents, around 115 (or 60.5) strongly believe that health practitioners have a responsibility to report ADRs. In spite of the requirement to report all suspected ADR cases encountered by healthcare practitioners, 96 (50.5%) of respondents agreed that they must confirm the ADR before reporting. Only 45.3% of the healthcare professionals surveyed agreed that reporting ADRs enhances patient safety, and only 29.5% thought that all suspected ADRs should be reported.

On the other side, 48.4% of respondents thought that ADR reporting trends identify generally safe medications, while 25.8% of survey participants argued that ADR reporting generates workload. Moreover, about 43.2% and 44.7% of the healthcare providers disagreed that ADR reporting is not important for the healthcare system and reporting of ADR affects patient's confidentiality issues, respectively. In addition, 11.6% and 21.1% of the respondents agreed that a single ADR report brings no difference and fear of legal liability affects ADR reporting, respectively.

### 3.6. Factors Associated with Attitude of Healthcare Providers towards ADR Reporting

The majority (53.7%) of the 190 healthcare professionals studied were found to have a positive attitude regarding ADR reporting. On the other hand, roughly 46.3% of study participants had a negative opinion of ADR reporting. The results of a multivariate logistic regression analysis showed that the attitude of healthcare practitioners toward ADR reporting was substantially correlated with professional distribution (*P* < 0.05). Therefore, compared to nurses, physicians and public health officials were more likely to have a negative attitude about ADR reporting (AOR = 1.76, CI: 0.29–10.48) and (AOR = 3.38, CI: 0.59–19.11), respectively (Table 6).

Years of service had a substantial effect on healthcare workers' attitudes toward ADR reporting. Healthcare providers having four to six years of work experience were 2.9 times more likely to report ADR cases to the concerned bodies as compared to healthcare providers having more than six years of work experience (AOR = 2.88, CI (0.65–5.47)).

### 3.7. Qualitative Result

Face-to-face, in-depth interviews were conducted with the five key informants. The key informants were hospital pharmacy director, chief clinical director, head of nursing service, public health officer, and psychiatry. They are between the ages of 32 and 41 and have 5–10 years of hospital experience. From the five key informants, three were males and two were females. The qualitative data from the key informant interviews were analyzed for content. The interview notes were read through and data were classified into categories. Once all the data had been sorted into categories, the categories were examined to determine the emerging themes.

### 3.8. Factors That Hindered ADR Reporting by Healthcare Practitioners

#### 3.8.1. Healthcare Practitioners Related Factors

The attitudes and knowledge of the healthcare practitioner were the themes that were found. The attitudes that were identified as impeding ADR reporting included fear of reporting an ADR because doing so is perceived as negligence or wrongdoing by healthcare professionals, expecting ADR reporting to be a burden, and thinking that information about ADRs has already been discovered and expressed in other studies or literature. They consequently think that reporting a new incidence of ADR is of little consequence because the suspected ADRs are identical to others mentioned in various literature. Another factor that influences the healthcare practitioners' reporting of ADRs is their inadequate awareness of the country's ADR reporting system and the locations where they could report.

A key informant reported that *“……some healthcare practitioners do not report ADRs for the reason that they are afraid that they will be responsible for the occurrence of ADR cases….”*

Another informant stated that *“……. submitting ADR cases to the concerned bodies is an extra work which is outside the duties given by the hospital…*.*”*

Also, *“….other criticizes the high amount of work in their hospital and others simply bypass……”*

Some of them believe that *“………ADR reporting is the work of the pharmacist and they should not be involved in it.”*

#### 3.8.2. Health Systems Factors

The inaccessibility of ADR reporting forms, the substantial workload placed on healthcare professionals, and the need to pay an additional fee to send an ADR report to the relevant agencies have all been noted as obstacles to ADR reporting by healthcare providers.

#### 3.8.3. ADR Reporting System in Ethiopia

For ADR reporting, efficient organization and structure are essential. The current system, according to some of the key informants, is unhealthy, decentralized, and not easily accessible to healthcare providers and facilities. The federal level housed the outgoing pharmacovigilance center. One of the key informants made clear the following.

*“…………… Ethiopia currently has just one national pharmacovigilance center. The center's decentralization will make its reporting forms and instructions more accessible. Face-to-face discussions are among the training activities that can be easily given at a cheap cost. Creating regional centers at university hospitals could assist and make it easier for healthcare providers to report ADRs.”*

## 4. Discussion

This study identified the willingness, attitude, practice, and associated factors of healthcare providers towards ADR reporting in Mizan Tepi University Teaching Hospital, Southwest Ethiopia. A significant proportion of healthcare professionals have ever reported ADR cases to the national regulatory agency. It was realized that the majority of study participants (53.7%) had a positive attitude regarding ADR reporting. The majority of study participants (81.6%) were willing to report adverse drug reactions to the appropriate authorities, while 18.4% of healthcare providers were not willing to report adverse drug reactions. Lack of an ADR reporting format in the healthcare facility and a lack of knowledge of the ADR reporting system were the causes of the unwillingness to submit ADRs. This result is in line with a study done in Nigeria, which discovered that the absence of an ADR reporting form and a formal reporting mechanism are factors influencing the unwillingness of health personnel to report ADRs [13].

The majorities of research participants in this study (57.9%) is knowledgeable about reporting ADRs and are aware of how to do so. This result is consistent with research done in other regions of Ethiopia [12] and Nigeria [13], which found that a substantially smaller percentage of healthcare professionals had inadequate knowledge of ADR. Our results, however, differ from those of a different country's study on ADR reporting knowledge, which revealed that health professionals have a low level of ADR reporting knowledge [19–21]. This divergence can be the result of differences in the study conditions as well as possible differences in the criteria used to determine the cutoff point. Healthcare professionals generally lack awareness of and inadequate expertise of ADR reporting, according to numerous researches [20–23]. The majority of study participants (57.9%) are aware of the proper channels to use when reporting ADR. In comparison to our research, only 48.2 percent, 34.2 percent, and 23.17 percent of healthcare practitioners in West Ethiopia [18], Amhara Region [20], and Southwest Ethiopia [24], respectively, had adequate knowledge on ADR reporting. These findings indicate that there are differences in understanding among Ethiopian health facilities, as well as access to ADR reporting information in our study location.

Regarding the healthcare professionals' reporting of ADRs, 86 (45.3%) of the respondents said they had dealt with ADR situations in the course of their careers. This result is higher than that of a study done in Ghana [25], but it is comparable to one done in Nigeria, where it was discovered that almost 60% of healthcare professionals had dealt with an ADR case in the course of their professional activity [13]. This discrepancy can be the result of a different study design, healthcare environment, period of time, or level of knowledge regarding the importance of disclosing a single adverse drug reaction. Moreover, half (53.7%) of healthcare professionals had two or more ADR cases reported to the authorities during the course of their professional careers. In the morning session, 60.5% of the healthcare experts had ever presented ADR cases. In comparison to other studies, the ADR reporting rate in this study is extremely low. It was made clear that healthcare professionals needed regular training, and the national regulatory agency needed to make the ADR reporting forms easily accessible. It is crucial to regularly monitor ADR cases and to bring up potential ADR situations during the morning session. According to various studies, providing access to the ADR reporting format and placing it in the provider's environment are crucial steps in encouraging healthcare professionals to report adverse events [26, 27].

The majority of the participants in this survey (53.7%) were found to have a positive attitude about ADR reporting. On the other hand, roughly 46.3% of study participants had a negative opinion of ADR reporting. These findings demonstrate that healthcare professionals' perceptions toward ADR reporting were largely favorable. The results of this study are less positive than those of studies carried out in other regions, including 66.3% in Nepal [28], 82.2% in South India [29], 60% in the Amhara Region of Ethiopia [20], and 75% in Southwest Ethiopia [24]. Lack of training on how to report and where to submit ADR cases, ignorance of the facility's ADR reporting form and system, and a lack of commitment by healthcare professionals to disclose ADR cases could all be contributing factors to the gap. Our findings also outshine those of a study done in West Ethiopia, which discovered that just 56 (42.1%) of healthcare professionals had a favorable view about ADR reporting.

The current study discovered that professional distribution and work experiences had a substantial impact on healthcare workers' attitudes toward ADR reporting. The results of a multivariate logistic regression analysis showed that the attitude of healthcare practitioners toward ADR reporting was substantially correlated with professional distribution (*P* < 0.05). Therefore, compared to nurses, physicians and public health officials were more likely to have a negative attitude about ADR reporting ((AOR = 1.76, CI: 0.29–10.48) and (AOR = 3.38, CI: 0.59–19.11), respectively). This is comparable to a study done in Northeast Ethiopia, which discovered that pharmacists were four times more likely to have adequate knowledge than nurses (93.1%), health authorities (90%), and doctors (86%) [30]. Additionally, comparable findings were found in other regions of Ethiopia, where nurses (*P*=0.001) and health officers (*P*=0.019) had less knowledge than pharmacists [19]. A study conducted at the University of Gondar in Ethiopia found that nursing students had a higher mean rank attitude score than that of pharmacy students and medical students (*P*=0.017) [31].

In this study, it was revealed that healthcare practitioners' opinions toward ADR reporting were substantially correlated with years of experience. When compared to healthcare professionals with more than six years of experience, those with four to six years of experience were 2.9 times more likely to report ADR cases to the relevant bodies (AOR = 2.88, CI (0.65–5.47)). This result is congruent with a study carried out in Southwest Nigeria, which discovered that health professionals with 1–5 years of PHC experience (68.8%) had a significantly more favorable attitude about ADR reporting than those with more than 5 years of experience (31.2%) (Pearson Chi-square (*X*^2^) = 5.24, *p*=0.02) [13]. Also, study done in Southwest Ethiopia showed that professional distribution was significantly associated with the attitude of healthcare providers toward counterfeit medicines [32]. Therefore, incorporating ADRs and pharmacovigilance themes into academic institutions and curricula where health disciplines are taught may be a significant use of formal educational channels to actively incorporate ADR/pharmacovigilance concept into the future careers of healthcare professionals, with the potential to significantly alter perception and attitude toward ADR reporting [27].

### 4.1. Limitations of the Study

A cross-sectional study design has a limitation in determining how healthcare providers behave when reporting ADRs because it just reveals what happened, not why it happened. Another limitation of the study was that the use of self-administered questionnaires may limit the accuracy of information that was obtained. However, this was mitigated by using qualitative methods to complement the data collected from the quantitative study and to provide more information on the health systems factors that affect the reporting of ADRs by health workers.

## 5. Conclusions

According to the study's findings, Mizan Tepi University Hospital healthcare providers rarely report adverse drug reactions. Most study participants were eager to alert the proper authorities about any adverse drug reactions. The health facility's lack of an ADR reporting format and the lack of knowledge about the ADR reporting system were the main deterrents to ADR reporting in the research area. The majority of healthcare professionals were found to view ADR reporting favorably. The attitude of healthcare providers toward ADR reporting was substantially correlated with professional distribution and work experiences. It is recommended that the health workers should receive training on ADR reporting. Health professionals should learn about the existence of the national program for reporting adverse drug reactions, the significance of reporting, who should report ADRs, and what information should be reported during the training.

Similarly, in order to improve the ADR reporting practices of the healthcare professionals and increase the ADR reporting load at the national level, the national regulatory body should collaborate with health facilities.

## Figures and Tables

**Table 1:** Sociodemographic profile of the healthcare providers' who participated in the study in Mizan Tepi University Teaching Hospital from August to September 2021.

Demographic variable		Frequency (%)
Sex	Male	109 (57.4)
	Female	81 (42.6)

Age	23–27	75 (39.5)
	28–32	79 (41.6)
	>32	36 (18.9)

Professional distribution	Pharmacy professional	32 (16.8)
	Physician	43 (22.6)
	Public health officer	5 (2.6%)
	Nurse	108 (56.8)
	Others^*∗*^	2 (1%)

Religion	Orthodox	85 (44.7%)
	Protestant	71 (37.4%)
	Catholic	8 (4.2%)
	Muslims	26 (13.7%)

Ethnicity	Bench	51 (26.8%)
	Oromo	57 (30%)
	Amhara	69 (36.3%)
	Keffa	5 (2.6%)
	Others^*∗∗*^	8 (4.2)

Year of service	1–3 years	110 (57.9)
	4-5 years	54 (28.4)
	≥6 years	26 (13.7)

^*∗*^Psychiatry and anesthesiologist; ^*∗∗*^Sheko, Shekka, Dawuro.

**Table 2:** Willingness of healthcare providers towards ADR reporting in Mizan Tepi University Teaching Hospital from August to September 2021.

Variables		*N* (%)
Have you willingness to report ADR	Yes	155 (81.6)
	No	35 (18.4)

If you say no to the above question, what are your reasons?	Lack of awareness about the ADR reporting system	9 (25.7)
	Lacks of ADR reporting format in your health facility	17 (48.6)
	Lacks of time	2 (5.7)
	Lacks of feedback	6 (17.1)
	Others	1 (2.9)

**Table 3:** Knowledge of healthcare providers towards ADR reporting in Mizan Tepi University Teaching Hospital from August to September 2021.

Variables		*N* (%)
Ever heard about existence of ADR reporting system in Ethiopia	Yes	139 (73.2)
	No	51 (26.8)

Knowledge about existence of national ADR monitoring guidelines	Yes	97 (51.1)
	No	93 (48.9)

Knowledge about how to report ADRs	Yes	110 (57.9)
	No	80 (42.1)

Organization responsible for monitoring ADR reports	Ministry of Health	73 (38.4)
	Ethiopian Food and Drug Administration (EFDA)	55 (29.9)
	Universities	12 (6.3)
	Ethiopian Public Health Institute (EPHI)	14 (7.4)
	Ethiopian Pharmaceutical Association	19 (10)
	I do not know	17 (8.9)

Knowledge about existence of ADR reporting form	Yes	119 (62.63)
	No	71 (37.37)

Professionals responsible in ADR reporting	Physicians	35 (18.4)
	Nurse	31 (16.4)
	Pharmacy professional	52 (27.4)
	All health professional	66 (34.7)
	I do not know	6 (3.2)

**Table 4:** Healthcare provider's practices towards ADR reporting in Mizan Tepi University Teaching Hospital from August to September 2021.

Variables		Frequency	Percentage (%)
Encounter ADRs during professional career	Yes	86	45.3
	No	104	54.7

Record ADR on patient medical records	Yes	53	61.6
	No	33	38.4

Ever report ADRs during your professional carrier	Yes	57	66.28
	No	29	33.72

Number of ADR cases reported during professional career	Only once	20	35.08
	2-3 times	31	54.39
	More than three times	6	10.53

Ever presented ADR cases at morning meeting	Yes	34	39.5
	No	52	60.5

**Table 5:** Attitude of healthcare providers towards ADR reporting in Mizan Tepi University Teaching Hospital from August to September 2021.

Variables	*N* (%)				
	Strongly agree	Agree	Neutral	Disagree	Strongly disagree
ADR reporting is duty of health professionals	115 (60.5)	65 (34.2)	3 (1.6)	5 (2.6)	2 (1.1)
ADRs need to be sure before reporting	79 (41.6)	96 (50.5)	4 (2.1)	10 (5.3)	1 (0.5)
ADR report improves patient's safety	81 (42.6)	86 (45.3)	11 (5.8)	11 (5.8)	1 (0.5)
All suspected ADRs should be reported	39 (20.5)	56 (29.5)	47 (24.7)	46 (24.2)	2 (1.1)
ADR reporting trends identify relatively safe drugs	45 (23.7)	92 (48.4)	33 (17.4)	17 (8.9)	3 (1.6)
ADR reporting creates workload	21 (11.1)	37 (19.5)	49 (25.8)	72 (37.9)	11 (5.8)
ADR reporting is not important for the healthcare system	12 (6.3)	23 (12.1)	18 (9.5)	82 (43.2)	55 (28.9)
Reporting of ADR affects patient's confidentiality issues	15 (7.9)	35 (18.4)	31 (16.3)	85 (44.7)	24 (12.6)
A single ADR report brings no difference	10 (5.3)	22 (11.6)	37 (19.5)	93 (48.9)	28 (14.7)
Fear of legal liability affects ADR reporting	32 (16.8)	40 (21.1)	37 (19.5)	66 (34.7)	15 (7.9)

**Table 6:** Factors associated with attitude of healthcare providers towards ADR reporting in Mizan Tepi University Teaching Hospital from August to September 2021.

Variables	Attitude category		AOR (95% CI)	*P* value
	Good attitude	Poor attitude		
Sex				
Male	59 (54.13%)	50 (45.87%)	1.00	
Female	43 (53.1%)	38 (46.9%)	1.05 (0.55–2.0)	0.884

Age				
23–27	40 (53.33%)	35 (46.67%)	0.68 (0.21–2.19)	0.518
28–32	42 (53.16%)	37 (46.84%)	1.00	
>32	20 (55.56%)	16 (44.44%)	0.62 (0.23–1.68)	0.353

Professional distribution				
Physician	23 (53.49%)	20 (46.51%)	1.76 (0.29–10.48)	0.536
Pharmacy professional	17 (53.12%)	15 (46.88%)	3.05 (1.49–18.73)	0.029^∗^
Public Health Officer	3 (60.00%)	2 (40.00%)	3.38 (0.59–19.11)	0.168
Nurse	58 (53.7%)	50 (46.3%)	1.00	

Years of service				
1–3 years	59 (53.64%)	51 (46.36%)	1.00	
4–6 years	29 (53.7%)	25 (46.3%)	2.88 (1.65–5.47)	0.044^*∗*^
≥6 years	14 (53.85%)	12 (46.15%)	1.19 (0.39–3.63)	0.756

^*∗*^ *P* value <0.05 was considered significant.

## Data Availability

The data used to support this study are included within the article.
